# Intake of Tibetan Hull-Less Barley is Associated with a Reduced Risk of Metabolic Related Syndrome in Rats Fed High-Fat-Sucrose Diets

**DOI:** 10.3390/nu6041635

**Published:** 2014-04-21

**Authors:** Lingxiao Gong, Lingyun Gong, Ying Zhang

**Affiliations:** 1 Department of Food Science and Nutrition, College of Biosystems Engineering and Food Science, Zhejiang University, Hangzhou 310058, China; E-Mail: samfy007@hotmail.com; 2 Department of Neurology, Huashan Hospital, Jingan 200040, Shanghai, China; E-Mail: 10913031@zju.edu.cn

**Keywords:** Tibetan hull-less barley (THB), whole grain, metabolic related syndrome, insulin resistance

## Abstract

The objective of this study was to assess the effects of whole grain Tibetan hull-less barley on metabolic related syndrome induced by high-fat-sucrose diets in rats. The diets were designed to reflect the dietary patterns of Chinese individuals (>30% energy fat) with refined wheat flour (HFS-W) or Tibetan hull-less barley (HFS-THB) as the main carbohydrate sources. Rats fed HFS-W had increased body weight, abdominal fat deposition, liver weight, liver fat deposition, triglyceride (TG), fasting blood glucose (FBG), serum fasting insulin (FINS), and homeostasis model assessment of insulin resistance (HOMA-IR) scores, and decreased low-density lipoprotein cholesterol (LDL-C) levels compared to rats fed a basal diet (BD). However, rats fed HFS-THB had reduced body weight gain, dyslipidemia, and insulin resistance. These findings indicate that whole Tibetan hull-less barley is a functional food that can reduce the prevalence of metabolic related syndrome induced by high-fat-sucrose diets.

## 1. Introduction

Metabolic syndrome (MS) comprises a cluster of pathologies: abdominal obesity, hyperglycemia (insulin resistance or impaired glucose tolerance and diabetes), dyslipidemia (hypertriglyceridemia and/or low HDL-cholesterol), and hypertension [1]. MS, which has become one of the major public-health problems worldwide, has been associated with increased risk of cardiovascular disease and type 2 diabetes [2].

MS is related to lifestyle, particularly to unbalanced energy-rich diets. Epidemiological studies have shown a strong negative relationship between the consumption of whole grains and the risk of developing MS [3]. In healthy middle-aged Tehranian adults, a higher intake of whole grains is associated with a 25% lower prevalence of impaired glucose tolerance [4]. Older adults with higher intakes of whole grain foods (*i.e.*, 2.9 servings/day) have a lower prevalence of MS (OR: 0.46; 95% CI: 0.27–0.79) compared to subjects with lower whole grain intakes (*i.e.*, <1 serving/day) [5]. Pereiar *et al.* reported that fasting insulin levels were 10% lower with whole grain diets than with refined grain diets [6]. Based on these studies, the 2005 Dietary Guidelines for Americans and Healthy People 2010 has recommended the consumption of at least three servings of whole grains per day [7,8].

Tibetan hull-less barley (THB) has been a staple food crop in Tibet for centuries. THB accounts for >97.7% of the total Tibetan barley varieties. The crop is cold- and drought-tolerant and can be cultivated at high altitudes; THB is the only crop that can be grown at >4200 m in the Tibetan Plateau. Extreme geographical conditions such as intense UV radiation, seasonal drought, and hypoxia contribute to lower crop yield but with plenty of secondary metabolites, including benzoic, cinnmaic acid derivatives, proanthocyanidins, flavonols and amino compounds [9,10]. Our previous research [11] has demonstrated that total soluble phenolic compounds and total antioxidant capacity of THB in the outer layers are more than two-fold higher than that of Tianjin hull-less barley (a variety grown in the plain). Moreover, among 164 Chinese barley cultivars, THB has the highest β-glucan content (approximately 8.62%) [12]. Over the past decade, THB has received considerable attention due to its health benefits. Epidemiological studies have reported a lower prevalence of hyperlipidemia and diabetes with the consumption of THB [13]. In a randomized double-blind trial, Xiong *et al.* [14] reported that the dietary intake of whole THB (W-THB) significantly reduced plasma cholesterol concentrations, serum triglyceride levels, and body weight in 227 dyslipidemic patients. Similarly, a recent study by Li *et al.* reported that W-THB significantly decreased postprandial blood glucose and insulin levels in healthy adults [15]. Altogether, these studies strongly support the idea that increasing the consumption of W-THB might be a useful dietary approach to lowering the risk of developing MS.

This study assessed the effects of W-THB in rats with MS. The main objectives of this study were to (1) investigate the effects of a hypo-caloric diet containing W-THB on rats with diet-induced MS and (2) evaluate whether MS is affected by whole grains, which contain nutrients and bioactive phytochemicals, but are highly caloric.

## 2. Methods

### 2.1. Animals

A total of 40 male Sprague-Dawley rats (4–5 weeks of age) weighing 170–210 g were obtained from Zhejiang Experimental Animal Center (Hangzhou, Zhejiang, China). The rats were housed (five per cage) in an air-conditioned room (23 ± 2 °C and 60% ± 10% relative humidity) with 12-h light and 12-h dark cycles. All experimental procedures were approved by the ethical committee for laboratory animals of Zhejiang University.

### 2.2. Diets

Experimental diets were designed to resemble a human high-fat-sucrose diet, in terms of the contribution of fat, protein, and carbohydrate to the total energy intake (Table 1).

**Table 1:** Composition and energy content of the experimental diets.

Components	Basal Diet (g/100 g)	High-Fat-Sucrose Diet (g/100 g)	
		Refined Wheat	W-THB
Whole wheat flour	30	-	-
Corn starch	36	-	-
Refined wheat flour	-	50.4	-
Whole Tibetan hull-less barley (W-THB)	-	-	50.4
Lard	-	10	10
York powder	-	5	5
Cholesterol	-	0.5	0.5
Sodium cholate	-	0.1	0.1
Sucrose	-	8	8
Cellulose	5	5	5
Soybean oil	1	-	-
Soybean meal	23	16	16
Vitamin mix	4	4	4
Mineral mix	1	1	1
Calculated energy			
Protein (%)	25.5	21.9	21.5
Carbohydrate (%)	63.2	42.7	42.0
Fat (%)	11.2	35.5	36.0
Energy (kcal/g)	3.60	3.94	3.97

The indexes for energy determination were 4.0 kcal/g (protein and carbohydrate) and 9.0 kcal/g (fat).

### 2.3. Experimental Procedures

Following a 3-day acclimation period, the rats were randomly assigned to one of three groups: (1) a basal diet group (BD; *n* = 10); (2) a high-fat-sucrose diet with refined wheat group (HFS-W; *n* = 15); or (3) a high-fat-sucrose diet with W-THB group (HFS-THB; *n* = 15). The rats were fed the experimental diets for 15 weeks. Body weights were monitored every week. Food intake was determined every week as following: presented dry food substrate by rejected dry food. The result was divided by 5 to take into account the presence of five rats per cage. Blood samples were taken on weeks 0, 4, 8, and 15 from 12-h fasted rats. These blood samples were used for plasma glucose and plasma insulin measurements. Plasma total cholesterol (TC), triglyceride (TG), high-density lipoprotein cholesterol (HDL-C), and low-density lipoprotein cholesterol (LDL-C) concentrations were determined in the endpoint plasma samples. At the end of the study, the rats were sacrificed with intraperitoneal chloral hydrate (100 g/L) following a 12-h fast. Epididymal fat, abdominal fat, and liver were removed, rinsed with phosphate-buffered saline, and weighed. The liver samples were stored at −70 °C.

### 2.4. Serum Lipid Profile

TC, TG, HDL-C, and LDL-C serum concentrations were determined in a Cobas 8000 modular chemistry analyzer (Roche Co., Mannheim, Germany).

### 2.5. Histological Observations of Hepatic Tissue

Sections of liver samples were fixed in a buffer solution containing 10% formalin and processed for paraffin embedding. Briefly, 4-μm sections were stained with hematoxylin-eosin and observed under light microscopy (Olympus BX41) with the magnifying power of 100×.

### 2.6. Hepatic Glycogen

Hepatic glycogen concentrations were determined with a Rat Hepatic Glycogen ELISA kit (Nanjingg Jiancheng Technology Co., Ltd., Nanjing, China).

### 2.7. Assessment of Insulin Resistance

Insulin resistance was assessed by the Homeostasis Model Assessment Insulin Resistance (HOME-IR) score, which is based on fasting plasma insulin and glucose concentrations according to the following Equation 1.

HOME-IR = [fasting insulin (mIU/L) × fasting glucose (mmol/L)]/22.5
(1)

Plasma insulin was measured with a Rat Insulin ELISA kit (Nanjingg Jiancheng Technology Co., Ltd., Nanjing, China) and plasma glucose was measured by the glucose oxidase method (Onetouch Ultra, Johnson & Johnson Co., New Brunswick, NJ, USA).

### 2.8. Statistical Analyses

All data were expressed as mean ± standard deviation. Simple linear regression analyses were performed to make adjustments for body weight. Data were analyzed by analysis of variance (ANOVA), followed by the Tukey’s *post hoc* test using the SPSS program (SPSS, Chicago, IL, USA). *P* < 0.05 was considered to be statistically significant.

## 3. Results

### 3.1. Feed Intake, and Body, Fat, and Tissue Weights

**Figure 1:**
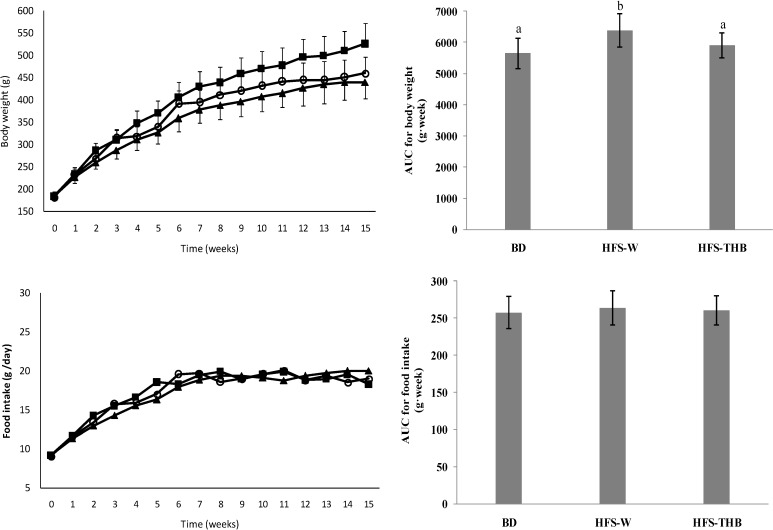
Body weight and food intake during the 15-week study. (▲) Basal diet intake group (BD); (**■**) High-fat-sucrose diet with refined wheat intake group (HFS-W); (○) High-fat-sucrose diet combined with whole Tibetan hull-less barley intake group (HFS-THB). Samples were analyzed using a one-way ANOVA followed by a Turkey post doc test. Evaluations were taken among three groups, a, b indicate statistical differences (*P* < 0.05), the same letters indicate no statistical differences (*P* > 0.05).

Throughout the feeding period, food intake did not differ among the three diet groups. Body weight increased during the study (Figure 1). The HFS-W rats gained more weight compared to the BD rats. In weeks 0 and 1, there were no differences in body weight among the groups. After week 2, the HFS-W rats had higher body weight than the BD rats (*P* < 0.05). However, the body weight of HFS-THB was not significantly different to that of BD (*P* > 0.05), except during weeks 3 and 6 (*P* = 0.06 and 0.032, respectively).

The area under curve (AUC) was calculated and analyzed. AUC for body weight in HFS-W was significantly higher than that in BD and HFS-THB (*P* < 0.05). However, there was no difference between BD and HFS-THB. AUC for food intake showed no differences among the three groups (*P* > 0.05).

The HFS-W group had higher tissue and fat absolute weights or fat relative weights than the BD group (*P* < 0.05; Table 2). However, the tissue relative weights did not differ between the BD and HFS-W groups. HFS-THB had lower liver absolute weight (−9.02%) and epididymal fat had lower absolute and relative weights (−37.98% and −29.59%, respectively) compared to HFS-W.

**Table 2:** Liver, kidney, epididymal fat, and perirenal fat weights at the end of the 15-week study.

Tissues	Basal Diet (*n* = 10)	High-Fat-Sucrose Diet	
		Refined Wheat (*n* = 14)	Whole THB (*n* = 14)
Liver (g)	11.2 ± 1.71 ^a^	13.3 ± 1.40 ^b^	12.1 ± 1.58 ^a,b^
Kidney (g)	2.78 ± 0.30 ^a^	3.23 ± 0.17 ^b^	3.28 ± 0.33 ^b^
Epididymal fat (g)	5.69 ± 2.13 ^a^	10.4 ± 4.90 ^b^	6.45 ± 1.56 ^a^
Perirenal fat (g)	1.90 ± 0.52 ^a^	3.90 ± 1.67 ^b^	3.19 ± 0.74 ^b^
Liver (% ^#^)	2.48 ± 0.31	2.57 ± 0.12	2.59 ± 0.36
Kidney (%)	0.621 ± 0.079 ^a^	0.626 ± 0.053 ^a^	0.689 ± 0.047 ^b^
Epididymal fat (%)	1.24 ± 0.35 ^a^	1.96 ± 0.79 ^b^	1.38 ± 0.29 ^a^
Perirenal fat (%)	0.420 ± 0.100 ^a^	0.735 ± 0.262 ^b^	0.683 ± 0.148 ^b^

Samples were analyzed using a one-way ANOVA followed by a Turkey post doc test. ^a,b^ within rows, between basal diet and high-fat-sucrose diet groups, indicate statistical differences at *P* < 0.05; ^#^ % of body weight.

Furthermore, the HFS-THB group had lower perirenal fat absolute and relative weights than the HFS-W group; however, these results were not significant (*P* > 0.05). The kidney relative weights of HFS-THB were significantly higher than those of HFS-W (*P* < 0.05).

### 3.2. Plasma Cholesterol, TG, and Lipoprotein Concentrations

The HFS-W group had significantly higher TC and lower HDL-C concentrations compared with the BD group (*P* < 0.05). There were no differences in TC or HDL-C between BD and HFS-THB (*P* > 0.05). Additionally, there were no differences among the three groups in plasma total cholesterol or LDL-C concentrations at the end of the 15-week study (Table 3).

**Table 3:** Plasma cholesterol, TG, and lipoproteins in rats at the end of the 15-week study (mmol/L).

Lipid Profiles	Basal Diet (*n* = 10)	High-Fat-Sucrose Diet	
		Refined Wheat (*n* = 14)	Whole THB (*n* = 14)
Total cholesterol	1.46 ± 0.32	1.47 ± 0.34	1.50 ± 0.23
TG	0.69 ± 0.24 ^a^	1.15 ± 0.23 ^b^	0.84 ± 0.12 ^a,b^
LDL-C	0.17 ± 0.03	0.14 ± 0.04	0.15 ± 0.03
HDL-C	1.39 ± 0.36 ^b^	1.02 ± 0.22 ^a^	1.40 ± 0.18 ^b^

Samples were analyzed using a one-way ANOVA followed by a Turkey post doc test. ^a,b^ within rows, between basal diet and high-fat-sucrose diet groups, indicate statistical differences at *P* < 0.05.

### 3.3. Histology of Liver

**Figure 2:**
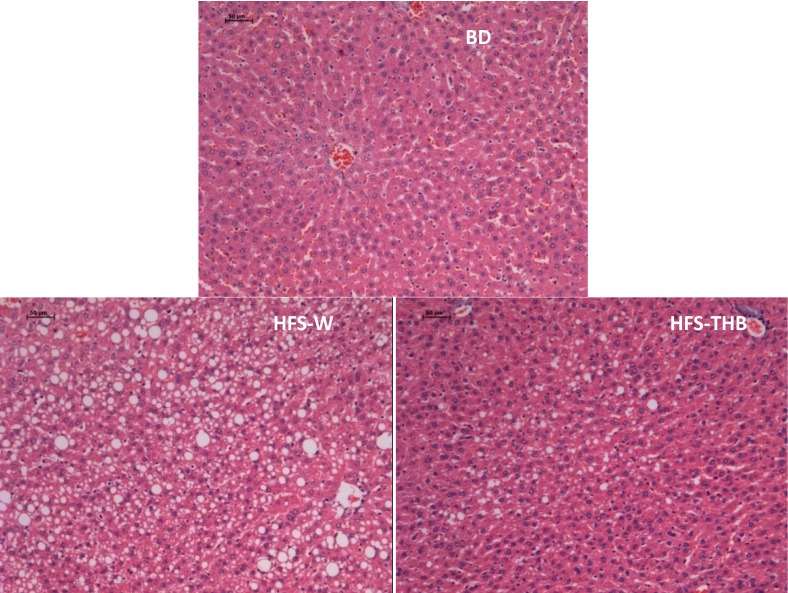
Histological features inon liver of in experimental rats (100×). Liver tissue of SD rats was stained with hematoxylin and exosin.

As shown in Figure 2, the examination of liver sections of HFS-W rats revealed the presence of a large number of circular lipid droplets. These lipid droplets were significantly reduced in size and number in HFS-THB rats, which may suggest that W-THB effectively inhibits lipid accumulation in liver tissue. However, it was not clear what the inclusions were. Oil Red staining to stain fat will be used to confirm the results in our future works.

### 3.4. Hepatic Glycogen

**Figure 3:**
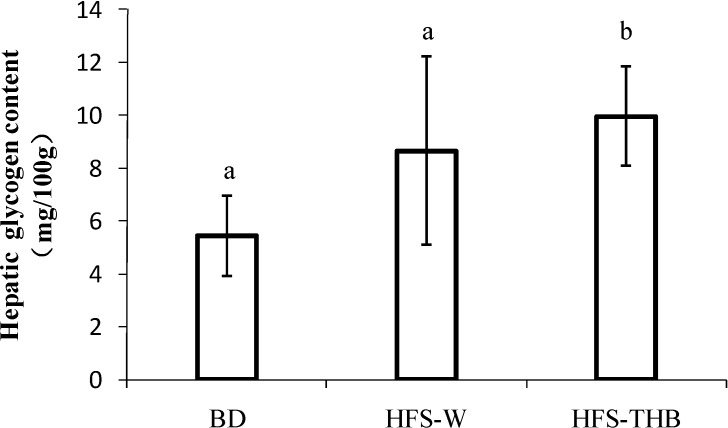
Hepatic glycogen in rats at the end of the 15-week study. Samples were analyzed using a one-way ANOVA followed by a Turkey post doc test. Evaluations were taken among three groups, a, b indicate statistical differences (*P* < 0.05), the same letters indicate no statistical differences (*P* > 0.05).

At the end of the 15-week study, hepatic glycogen was significantly higher in the HFS-TB group (15.0 ± 6.58 mg/100 g) than in the BD group (5.45 ± 1.51 mg/100 g; *P* < 0.05). Hepatic glycogen in HFS-W was higher compared to that in BD; however, this result was not significant (10.3 ± 2.75 mg/100g; *P* > 0.05) (Shown in Figure 3).

### 3.5. Insulin Resistance, Plasma Glucose, and Plasma Insulin

**Figure 4:**
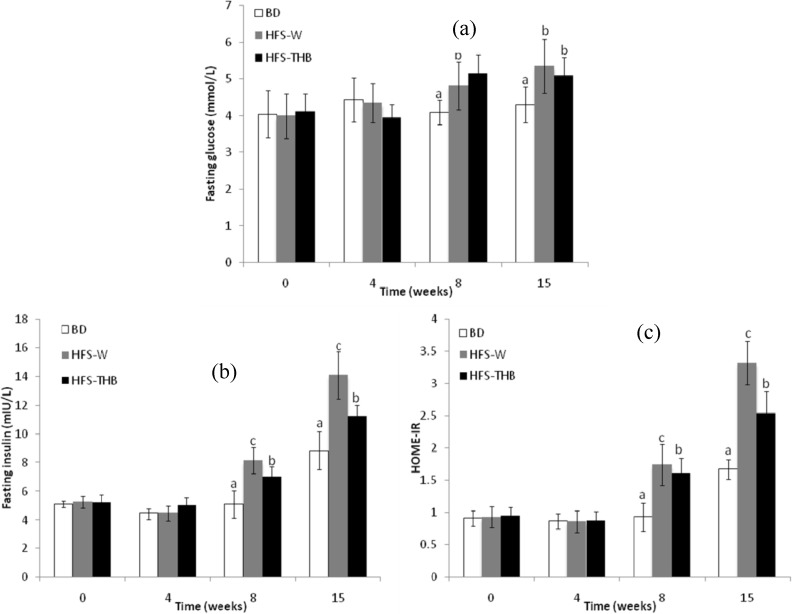
Concentration of plasma (**a**) glucose and (**b**) insulin, and (**c**) insulin resistance of rats during the 15-week study. BD, basal diet; HFS-W, high-fat-sucrose refined wheat diet; HFS-THB, high-fat-sucrose whole Tibetan hull-less barley diet. Samples were analyzed using a one-way ANOVA followed by a Turkey post doc test Evaluations were taken among three groups, a, b, c indicate statistical differences (*P* < 0.05), the same letters indicate no statistical differences (*P* > 0.05).

The changes in fasting blood glucose (FBG), serum fasting insulin (FINS), and homeostasis model assessment of insulin resistance (HOMA-IR) scores are shown in Figure 4. FBG, FINS, and HOMA-IR were not different among the three groups at weeks 0 and 4. However, after week 8, FBG, FINS, and HOMA-IR of HFS-W and HFS-THB were significantly higher than those of BD (*P* < 0.05).

At week 8, FBG of HFS-W and HFS-THB was significantly higher (+17.6% and +25.7%, respectively) than FBG of BD (4.09 ± 0.33 mmol/L). In weeks 8–15, FBG did not change in the three experiment groups; however, FBG remained higher in HFS-W and HFS-THB than in BD. There were no significant differences between HFS-W and HFS-THB during the experiment (*P* > 0.05).

After week 8, FINS was higher in HFS-W and HFS-THB than in BD. At week 8, FINS of BD, HFS-W, and HFS-THB was 5.09 ± 0.95 mIU/L, 8.13 ± 0.93 mIU/L, and 7.04 ± 0.67 mIU/L, respectively. Therefore, HFS-THB had a FINS value that was 13.41% lower than that of HFS-W. The three groups had higher FINS at week 15 than at week 8: FINS of BD, HFS-W, and HFS-THB increased by 73.28%, 73.31%, and 59.38%, respectively. However, FINS was significantly higher in the HFS-W and HFS-THB groups than in the BD group after 15 weeks. FINS of HFS-W and HFS-THB were 59.75% and 27.21% higher than that of BD (8.82 ± 1.32 mIU/L), respectively. In addition, FINS of HFS-THB was significantly lower compared with that of HFS-W, which was reduced by approximately 20.37% from a mean value of 14.09 ± 1.64 mIU/L.

Overall, the changes in HOME-IR scores were similar to those of FINS. At week 8, HOME-IR of HFS-W and HFS-THB was 0.87 and 0.73 times higher, respectively, than HOME-IR of BD. On the other hand, HOME-IR of HFS-THB was 7.47% lower than that of HFS-W (*P* < 0.05). At the end of the 15-week study, HOME-IR of BD, HFS-W, and HFS-THB was significantly higher than that at the beginning of this study, which indicated that insulin sensitivity was affected by rat growth. The HOME-IR scores of BD, HFS-M, and HFS-THB at week 15 were approximately 0.83, 2.57, and 1.67 times higher than those at week 0, respectively. The HOME-IR scores of HFS-W and HFS-THB increased more than those of BD. Moreover, at week 15, the HOME-IR scores of HFS-W and HFS-THB were 0.98 and 0.52 times higher than those of BD, respectively. However, the consumption of W-THB improved insulin sensitivity. The HOME-IR scores of HFS-THB were 23.4% lower than those of HFS-W (*P* < 0.05).

As shown in Table 4, once adjusted for body weight, the fasting insulin differed only at week 15 among the three groups. Further studies are necessary to clarify the relative contributions of weight gain to insulin secretion, insulin sensitivity, and the interaction between them.

**Table 4:** Difference in fasting insulin among three groups after adjustment for body weight at week 15.

Group	*P* Value		
	BD	HFS-W	HFS-THB
BD	-	0.035	0.043
HFS-W		-	0.069
HFS-THB			-

Samples were analyzed using linear regression analyses.

## 4. Discussion

The results of this study revealed that the long-term consumption of W-THB could ameliorate metabolic related syndrome in rats fed high fat and sucrose diets.

Animal models have been used to study metabolic related syndrome in humans; several studies have focused on the effects of whole grains on the pathogenesis of metabolic related syndrome [3,16]. The experimental design of these studies varied considerably contributing to different results. Some studies reported that whole grains improve fasting glucose levels, fasting and acute insulin concentrations, and glucose responses [17,18,19]. However, other studies have failed to show any effect of whole grains on glucose metabolism [20,21]. Additionally, some studies reported decreased total cholesterol, LDL-C, and increased HDL-C from whole grain-rich diets [17,22]. However, Andersson *et al.* [23] and Brownlee *et al.* reported no significant effects of whole grains on lipid levels [20].

In 2005, the International Diabetes Federation (IDF) outlined the clinical diagnosis criteria for MS: central obesity and any two of the following four factors, high TG levels, low HDL-C levels, high blood pressure, or high fasting plasma glucose [24]. The SD rat model is commonly used to assess diet-induced metabolic related syndrome [25]. The World Health Organization (WHO) recommends that fat intakes and refined carbohydrate intakes should be <30% and <10%, respectively, of the total calories.

In this study, a high-fat-sucrose diet with refined wheat flour had 21.5%–21.9% protein, 35.3%–36.5% fat, and 42.0%–42.7% carbohydrates, which has a similar composition to that of Chinese diets [26]. The results revealed that high-fat-sucrose diets can induce abdominal obesity, dyslipidemia, hyperglycemia, and insulin resistance in rats, consistent with the results of previous studies [27,28]. Refined wheat flour is the starchy endosperm left after removing the pericarp seed coat, aleurone, and germ (including large amounts of fiber, micronutrients, protein and phytochemicals). The nutrient composition of refined wheat is different to that of the original whole grain seed [29].

To the author’s knowledge, this is the first study that assessed the effects of W-THB on diet-induced metabolic related syndrome in an animal model. The consumption of W-THB ameliorated the development of dyslipidemia and insulin resistance and was useful in weight control.

The HFS-W rats had higher body weight and visceral fat than the HFS-THB rats. Similar clinical findings have been found in obese men by Koh-Banerjee *et al.*, who reported that whole grains are more satiating than refined grains [30]. Additionally, W-THB reduced visceral fat. It is noteworthy that, in the present study, W-THB significantly decreased the epididymal fat absolute weight of HFS-W rats by 37.98%; however, the perirenal fat absolute weight decreased by 18.20%. The reason for the different effects of W-THB on epididymal fat and perirenal fat is unknown.

Lipid droplets represent an inert storage pool of TG [31]. The high-fat-sucrose diet resulted in an accumulation of lipids in hepatocytes, which contributes to liver damage, such as non-alcoholic fatty liver disease. The number of lipid droplets in the liver of HFS-THB rats was lower in comparison to that of HFS-W rats. Therefore, the consumption of W-THB might improve serum lipid profiles by suppressing the accumulation of body fat even with the consumption of high-fat diets. Adiposity and insulin resistance are hallmarks of MS; the latter may be closely tied to low grade inflammatory responses to diet [32]. The results of this study revealed that there were no differences in the glycemic parameters among groups after being fed for 4 weeks; however, FBG, FINS, and HOMA-IR of the HFS groups were higher compared to those of BD. The increase in FBG, FINS, and HOMA-IR from week 0 to week 15 indicated that insulin sensitivity was progressively impaired by diet. Furthermore, this study revealed an improvement in insulin resistance (lower HOMA-IR scores) with the consumption of W-THB for 8 to 15 weeks. The consumption of W-THB had beneficial effects on glucose and insulin responses. The results are consistent with the findings obtained from animal and clinical trials, which revealed that the consumption of whole grains may protect against type 2 diabetes by improving glucose control and insulin sensitivity [6,33].

HFS significantly increased hepatic glycogen levels. In the liver, glucose is produced by glycogenolysis and gluconeogenesis. Free fatty acids increase gluconeogenesis and suppress glycogenolysis by activating glycogen synthase and inhibiting glycogen phosphorylase [34,35]. On the other hand, hyperinsulinemia suppresses hepatic gluconeogenesis to impair glucose storage in liver [36]. Previous studies have suggested that a compensatory reduction in glycogenolysis ameliorates the FFA-induced increase in gluconeogenesis from elevating hepatic glucose production. This is defined as “hepatic autoregulation” [37]. HOMA-IR scores revealed that the consumption of W-THB improved insulin sensitivity and reduced FINS in the HFS-THB group relative to the HFS-W group. This may be the reason responsible for the lower hepatic glycogen content in HFS-W rats.

The precise mechanisms by which W-THB improves metabolic related syndrome in rats were not explored in this study. Energy intake did not differ among the groups; therefore, the reduced body weight gain in the HFS-THB group was not attributed to a reduced energy intake. The effect of W-THB on diet-induced metabolic related syndrome might be attributed to the synergistic effects of its nutrients and bioactive compounds. From 164 barley cultivars originating in China, THB has the highest β-glucan content [23]. Moreover, the β-glucan content of major Tibetan cultivars is higher than that of Harrington, CDC Richard, and CDC Buck in Canada and is significantly higher than that of wheat and rice [38].

Consumption of a diet high in β-glucan ameliorates fatty liver and insulin resistance in mice fed high-fat diets. The β-glucan diet promoted hepatic insulin signaling by decreasing serine phosphorylation of insulin receptor substrate 1, activating Akt, and decreasing mRNA levels of glucose-6-phosphatase and phosphoenolpyruvate carboxykinase [39].

Researchers have reported that components of barley other than β-glucan have beneficial effects on human health. Qian *et al.* treated hyperlipidemic SD rats with W-THB bran oil. The bran oil group had significant reductions in TC, TG, and LDL-C and had a significant increase in HDL-C. Bran oil has a high content of linoleic acid (75.08% of the total fatty acids) [40]. Linoleic acid and other *n*-6 fatty acids decrease serum TC and LDL-C levels [41,42]. Additionally, W-THB has a number of protective compounds, *i.e.*, insoluble fiber, which increases transit time and fecal bulk; Zn, Se, and nicotinic acid, which participate in hormonal activation and synthesis; and polyphenols, which regulate gene expression [29].

## 5. Conclusions

By replacing refined wheat flour in a high-fat-sucrose diet with W-THB, the prevalence rate of metabolic syndrome in rats decreased. These findings indicate that W-THB improves metabolic syndrome induced by high-fat-sucrose diets. However, the mechanisms by which W-THB affects the pathogenesis of metabolic syndrome are unknown and require further investigation.
